# Evaluation of periodontal health among old Indian patients with glaucoma and chronic tonsillitis

**DOI:** 10.6026/9732063002001136

**Published:** 2024-09-30

**Authors:** Akbar Naqvi, Jameel Qazi, Khushtar Haider, Sushil Ojha, Anjali A, Santosh Kumar

**Affiliations:** 1 Department of Dentistry, Hamdard Institute of Medical Sciences and Research, New Delhi, India; 2 Department of Ophthalmology, Government Doon Medical College and Associated Doon Hospital Dehradun Uttarakhand, India; 3 Oral & Maxillofacial Pathology & Microbiology, Buddha Dental College and Hospital, Patna, Bihar, India; 4 Department of Dentistry, Madhubani medical College Bihar, India

**Keywords:** Periodontal health, glaucoma, chronic tonsillitis, aging adults, old population

## Abstract

Periodontitis, marked by the deterioration of the tissues bordering the surfaces of the teeth, is one of the most prevalent diseases. The presence of pathogenic bacteria, also known as "periopathogens" in the gingival sulcus region is the main cause of periodontitis. Analyzing the relationship between ocular disorders, chronic tonsillitis and periodontitis has been the subject of few investigations. The aim of this study was to evaluate the association between periodontitis, glaucoma and chronic tonsillitis. This study evaluated periodontal health in glaucoma and chronic tonsillitis patients among ageing adults and the elderly population. It was a case-control study in which there was an evaluation of the correlation between periodontitis glaucoma and chronic tonsillitis. 120 clinically diagnosed cases of glaucoma and 120 clinically healthy individuals were taken as controls. 123 cases of clinically diagnosed cases of chronic tonsillitis were taken as controls. In every clinically diagnosed case, there was a thorough periodontal examination by measuring the following parameters. PI: periodontal index. CAL: Clinical attachment loss. BI: Bleeding index. There was a collection of biofilm specimens from the buccal surface of the maxillary first molar, second molar and mandibular anterior for evaluation of colonies of Streptococcus mutans. The specimens were cultured in agar plates. The CFU/ml was calculated. Logistic regression analysis revealed that there was statistically significant difference in values of PI (p<0.001) indicating poor periodontal health in glaucoma patients and chronic tonsillitis patients. There was statistically significant difference in values of BI (p<0.001), indicating increased bleeding from gums in glaucoma patients and chronic tonsillitis patients. In our study there was statistically significant difference in values of CAL indicating increased attachment loss in glaucoma patients and chronic tonsillitis patients. There was statistically significant difference in values of CFUs of Streptococcus mutans in plaque samples indicating increased CFUs of Streptococcus mutans in chronic tonsillitis and glaucoma patients. This study demonstrated the link between periodontitis glaucoma and chronic tonsillitis and offered a potential explanation. To solve this study's shortcomings, more research would be required. Examining individuals with chronic tonsillitis and glaucoma requires determining the oral cavity's state and computing dental indices, of which the periodontal index is crucial. Recommending extensive therapy for patients suffering from periodontitis, glaucoma, and chronic tonsillitis is imperative by ophthalmologists, ENT professionals and periodontists.

## Background:

One of the most common illnesses is periodontitis, which is characterized by the breakdown of the tissues that line the gums [1, 2].The primary cause of periodontitis is the existence of pathogenic microorganisms, sometimes referred to as "periopathogens" in the area of the gingival sulcus [3, 4]. Proteolytic enzymes as well as lipopolysaccharides operate as a mediating mechanism for the primary etiological components of chronic inflammation of periodontium, which include tobacco use, nutritional deficiency an inflammatory reaction, and insufficient oral hygiene [4, 6]. Furthermore, the host's immunological response is mediated by genetic and environmental variables. Periodontal inflammatory conditions, a breakdown of bone and periodontal adhesion resulting in loosening of teeth and periodontal pocket development, gingival inflammatory processes, haemorrhage and/or recession are the features of periodontitis [7, 8]. About 60 and 70 percent of elderly persons in the US had periodontitis. Numerous large-scale population studies were conducted in response to its rising incidence to determine whether periodontitis and systemic health conditions that are common in older adults are related [9, 10].

A common consequence of glaucoma, a chronic deteriorating ocular illness, is vision loss due to impairment of the optic nerve. It is the second most common cause of blindness worldwide and is an optic neuropathy [11-13]. Based on the condition of the iridocorneal outflow angle at the site of the aqueous humour discharge from the eye and the way it correlates with the intraocular pressure, subcategories of glaucoma are identified. Open-angle glaucoma is the most common variant; it is painless, advances slowly, and often results in blindness if treatment is not received. The drainage angle gradually closes in closed-angle glaucoma as the peripheral iris bows forward either abruptly or gradually, whereas in open-angle glaucoma the iridocorneal angle promotes unrestricted fluid outflow within the eyes [14, 15]. Abrupt oblique ophthalmosis is characterized by a middilated pupil, redness of the eye, intense discomfort in the eye, and nausea. Reduced vision loss from glaucoma is irreversible and is caused by the loss of axons in the ganglion cell layer as a result of a narrower neuro-retinal margin on the optic nerve [11-14].

Both glaucoma and periodontitis are more common as people age and are influenced by systemic variables such as body mass index (BMI), diabetes, smoking, drinking, and alcohol use [15,16]. Aging adults are normally belonging to 40 and above years. Analyzing the relationship between ocular disorders and periodontitis has been the subject of a few investigations [17-20]. Inflammatory condition of the tonsils, or tonsillitis, is a frequent illness that accounts for 1.3 percent of visits to the hospital. Mostly caused by an infection caused by bacteria or viruses, it manifests as throat discomfort when it's simple [21-23]. A clinical diagnosis of chronic tonsillitis is made. It might be challenging to distinguish between bacterial as well as viral causes, but doing so is essential to avoiding the abuse of antibiotics. Most cases of tonsillitis are caused by an infection, which can be bacterial or viral [24, 25]. Additionally, there is a chance that perio pathogens will infect the tonsils and cause tonsillitis. Studies assessing the periodontal health of elderly and geriatric individuals with glaucoma and chronic tonsillitis are extremely scarce. Thus, the aim of the study was to assess periodontal health in individuals with glaucoma and chronic tonsillitis among elderly (aged 40 to 70).

## Materials & Methods:

In this cross-sectional study, there was evaluation of periodontal health in 363 subjects among aging adults and the old population (40-70 years age group). Category A consists of 120 clinically diagnosed cases of glaucoma and Category B- 120 clinically healthy individuals and Category C- 123 cases of clinically diagnosed cases of chronic tonsillitis. The Institutional Review Board (IRB) HIMSR/2024/234 granted ethical approval for this study. Clinically diagnosed cases of glaucoma, chronic tonsillitis and normal healthy subjects aged between 40 to 70 years were included. Individuals having systemic disease were excluded from the study. The duration of the study is 5 months.

## Glaucoma Diagnosis:

Glaucoma can be diagnosed using various methods, including intraocular pressure measurement or tonometry, imaging studies, dilated eye exams, visual assessments, corneal thickness determination, and gonioscopy. These methods help assess for optic nerve injury, visually impaired regions, corneal thickness, and drainage angle.

## Chronic Tonsillitis Diagnosis:

Physical examination consists of examining the neck with a lit device and the nose and ears, which are other possible infection sites. Looking for the scarlatina rash, which has been linked to strep throat in some cases by touching the neck gently to look for enlarged lymph nodes or glands? It is done by using a stethoscope to listen to research participants' respirations and examining the spleen for enlargement to rule out mononucleosis, which irritates the tonsils.

## Throat Swab:

In this easy test, the participant's throat is probed with a sterile swab from the back to collect a specimen of secretions. The sample was examined for streptococcal bacteria in a laboratory.

## Total Cell Count (CBC):

Your doctor may request a minimal amount of participant blood for a complete blood cell count (CBC). This test yields a count of the various blood cell types. The profile of high, normal, and below-normal values might help determine whether a viral or bacterial agent is more likely to be the source of an infection. For the diagnosis of strep throat, a CBC is rarely required. To help identify the aetiology of tonsillitis, the CBC may be required if the strep throat lab test comes negative. In every clinically diagnosed case, there was a thorough periodontal examination by measuring the following parameters -PI: periodontal index, CAL: Clinical attachment loss and BI: Bleeding index. There was a collection of biofilm specimens from the buccal surface of the maxillary first molar, second molar and mandibular anterior for evaluation of colonies of Streptococcus mutans. The specimens were cultured in agar plates. The CFU/ml was calculated.

## Statistical analysis:

All of the information was gathered and recorded in an MS Excel document. The SPSS version 21 was used for statistical analysis. ANOVA test and chi-square test were used for statistical analysis. P value ≤ 0.05 was considered statistically significant.

## Results:

The mean age of study participants in category A, category B and category C was 56.24±1. 4 years, 54.15± 1.4 years and 54.27±1.7 years respectively. The M:F in category A, category B and category C was 1.2:1, 1.1:1 and 1.1:1 respectively. There was no statistically significant difference in age and gender ratio among study participants of all three categories. (p>0.05), as seen in Table 1.

The Periodontal Index (PI) of study participants in Category A, category B and Category C was 4.7± 0.14, 2.4± 0.21 and 4.1± 0.16 respectively. The values of PI were greater in both category A and category C patients as compared to category B patients. Logistic regression analysis showed that there was statistically significant difference in values of PI (p<0.001) indicating poor periodontal health in glaucoma patients and chronic tonsillitis patients as compared to normal healthy subjects, as seen in Table 2.

## Pi- periodontal index:

The Bleeding Index (BI) of study participants in category A, category B and category C was 4.1± 0.11,2.1 ± 0.17 and 3.7 ± 0.15 respectively. Logistic regression analysis showed that there was statistically significant difference in values of BI (p<0.001) indicating increased bleeding from gums in glaucoma patients and chronic tonsillitis patients as compared to healthy subjects as shown in Table 3.

## Bi-bleeding index:

The Clinical Attachment loss (CAL) of study participants in category A, category B, category C was 5.2 ± 0.13, 3.1 ± 0.19 and 4.9 ± 0.15 respectively. Logistic regression analysis showed that there was statistically significant difference in values of CAL (p<0.001) indicating increased attachment loss in glaucoma patients and chronic tonsillitis patients as compared to normal healthy subjects, as seen in Table 4.

The colony-forming units (CFUs) of Streptococcus mutans in study participants in category A, category B and category C was <106 CFU/ml, 105-106 CFU/ml and 105-106 CFU/ml respectively. Logistic regression analysis showed that there was statistically significant difference in values of CFUs of Streptococcus mutans (p<0.001) indicating increased CFUs of Streptococcus mutans in plaque in glaucoma patients and chronic tonsillitis patients compared to healthy subjects as shown in Table 5, Table 6.

## Discussion:

This study was conducted to evaluate periodontal health in glaucoma patients and chronic tonsillitis patients in aging adults and old population of 40-70 years old. There was statistically significant difference in values of PI indicating poor periodontal health in glaucoma patients and chronic tonsillitis patients. There was statistically significant difference in values of BI indicating increased bleeding from gums in glaucoma patients and chronic tonsillitis patients. In our study there was statistically significant difference in values of CAL indicating increased attachment loss in glaucoma patients and chronic tonsillitis patients. There was statistically significant difference in values of CFUs of Streptococcus mutans in plaque samples indicating increased CFUs of Streptococcus mutans in chronic tonsillitis and glaucoma patients. Oxidative stress may share a pathophysiological mechanism across the two disorders. An imbalance between the generation of extremely reactive chemical species, such as reactive oxygen/nitrogen species (ROS/RNS) and the antioxidant mechanism is known as oxidative stress [18, 19]. According to earlier research, individuals with chronic periodontitis have reduced antioxidant capacity compared to controls, suggesting bacteria may be connected to oxidative stress in those who have the condition [20- 25].Although it has been studied for decades, the mechanism of oxidative stress's role in periodontitis is still unclear [16, 17]. According to certain research, patients with periodontitis have fewer leukocytes overall and have lower levels of oxidative activity [18]. Patients with periodontitis had larger concentrations of free radicals in their leukocytes, according to other research [19]. These conflicting results may be explained by the constantly changing pathogenesis of periodontitis and the many forms of the disease [20]. Oxidative stress has an unclear mechanism, and antioxidants can influence several processes unrelated to the production or activity of free radicals [21]. Although oxidative stress and periodontitis have been linked in several studies, it is unclear how oxidative stress plays a role in the pathogenesis of periodontitis [22]. It is believed that the pathophysiology of several diseases is connected to oxidative stress in periodontitis. Oxidative stress is a common cause of inflammatory illnesses.

Reports have examined the relationship between periodontal conditions and oxidative damage in systemic disorders [23, 24]. The relationship between chronic periodontitis and acute coronary syndrome was examined by Nguyen *et al.* [24]. According to a different study, patients with chronic periodontitis and rheumatoid arthritis had decreased antioxidant capacity and significantly greater levels of reactive oxygen species indicators in their plasma [25]. Numerous eye conditions, including diabetic retinopathy, glaucoma, and ocular surface abnormalities, have been linked to oxidative stress [16-18]. Patients with primary open-angle glaucoma also showed signs of oxidative damage in their DNA [19].

A complex immuno-pathological process known as chronic tonsillitis plays a role in the pathology associated with tonsillitis. Consequently, this tonsillitis-associated disease exacerbates and intensates the chronic tonsillitis course [24, 25]. Data regarding the potential impact of the oropharyngeal locus of focused persistent illness on the entire body may be found in the literature. Periodontal pockets, which are created when inflammation occurs in periodontal tissues, are one of these sites that might prolong the healing process of chronic tonsillitis and preserve body sensitivity [21-25]. Periodontal pockets' highly harmful microorganisms release bacterial endotoxins, which boost the body's immune system. The entire organism becomes intoxicated and sensitized by bacteria and the waste materials they produce. It becomes a vicious cycle that is very challenging to escape. A study by Kryukov *et al.* [25], Assessed how long a chronic tonsillitis course is affected by an ongoing process of inflammation in periodontal disease. Examined were seventy patients with recurrent tonsillitis. In collaboration with a dentist and perio-dontist, an evaluation of the condition of the oral cavity was conducted. Based on the findings, all patients suffering from persistent tonsillitis were split into two groups: those with and those without periodontal illnesses. Periodontal pockets have been reported to contain highly pathogenic flora in people with periodontitis.

Innate immunity and chronic inflammation are linked to glaucoma and periodontitis; innate immunity is recognized to contribute to several chronic inflammatory illnesses [19]. By activating toll-like receptors (TLRs), periodontitis triggers an innate immune response and an inflammatory response. The chronic interaction with fungal spores, viruses, bacteria and pollutants in the environment can induce the release of cytokines like TNF-α, IL-1, IL-6 and IL-17 from oral epithelial cells. These cytokines attract certain immune cells like neutrophils, T cells, eosinophils, macrophages, dendritic cells and natural killer cells that may be important in the pathophysiology of chronic inflammation of periodontium and activate several inflammatory pathways [21, 22]. Elevated intraocular pressure is a characteristic of glaucoma, but a more precise definition would be that it is a neuro-inflammatory as well as neurodegenerative illness that causes the retinal ganglion cells to degenerate [23]. Elevated intraocular pressure in glaucoma induces an innate immune response that often includes the invasion of monocytes and macrophages, along with resident immune cells such as microglia [24]. The microglia-triggered neuro-inflammatory response is thought to be a major factor in the pathogenesis of glaucoma. Proinflammatory substances like TNF-α, IL-6 and IL-1 are secreted by activated microglia, which also use TLRs to initiate innate immune responses [25]. Understanding the connection between glaucoma and periodontitis requires an understanding of inflammation and innate immunity.

The smaller population selected in the study is the limitation of the study and Further studies should try to enroll a more diverse population at baseline and use prospective investigation to confirm these relations. Lastly, the cross-sectional study design also poses a major limitation since it only allows the tracking of correlations rather than effects from one variable to another. More longitudinal researches with higher and randomly chosen samples are warranted to replicate the results and to clarify the processes.

## Conclusion:

Data shows that poor periodontal health in patients with glaucoma and chronic tonsillitis among ageing adults and the old population. Examining individuals with chronic tonsillitis and glaucoma requires proper assessment of the periodontal health also and taking proper steps of management of periodontal health.

## Figures and Tables

**Table 1:** Demographic details of study participants in category A, category B and category C

	**Age (mean±SD) years**	**M:F**
Category A	56.24±1.4	1.2:1
Category B	54.15±1.4	1.1:1
Category C	14.27±1.7	1.1:1

**Table 2:** Analysis of values of PI in patients of glaucoma (category A), chronic tonsillitis (Category C) and normal healthy age and gender matched controls (category B)

	**Mean PI**	**SD**
Category A	4.7	0.14
Category B	2.4	0.21
Category C	4.1	0.16
P value	<0.001	

**Table 3:** Analysis of values of BI in patients of glaucoma (category A), chronic tonsillitis (Category C) and normal healthy age and gender matched controls (category B)

	**Mean BI**	**SD**
Category A	4.1	0.11
Category B	2.1	0.17
Category C	3.7	0.15
P value	<0.001	

**Table 4:** Analysis of values of CAL in in patients of glaucoma (category A), chronic tonsillitis (Category C) and normal healthy age and gender matched controls (category B)

	**Mean CAL (mm)**	**SD**
Category A	5.2	0.13
Category B	3.1	0.19
Category C	4.9	0.15
P value	<0.001	
CAL- Clinical Attachment Loss

**Table 5:** Analysis of values of CFUs of Streptococcus mutans in patients of glaucoma (category A), chronic tonsillitis (Category C) and normal healthy age and gender matched controls (category B)

	**Mean CFU/ml**
Category A	<10^6^
Category B	10^5^-10^6^
Category C	10^5^-10^6^
P value	>0.001
CFU- Colony forming units

**Table 6:** Logistic regression analysis for parameters of periodontal health like PI, BI, CAL and Streptococcus mutans CFUs in three categories

	**Category A**	**Category B**	**Category C**
PI (mean±SD)	4.7± 0.14	2.4±0.21	4.1±0.16
Adjusted PR (95% CI)	1.95 (1.80 - 2.10)	Reference	1.70 (1.58-1.82)
		p<0.001	
BI (mean±SD)	4.1±0.11	2.1±0.17	3.7±0.15
Adjusted PR (95% CI)	1.95 (1.76-2.04)	Reference	1.76 (1.64-1.88)
		p<0.001	
CAL (mean±SD) mm	5.2 ± 0.13	3.1 ± 0.19	4.9 ± 0.15
Adjusted PR (95% CI)	1.67 (1.33-1.96)	Reference	1.58 (1.41-1.87)
		p<0.001	
Streptococcus mutans (Mean CFU/ml)	<10^6^	10^5^-10^6^	10^5^-10^6^
Adjusted PR (95% CI)	1.32 (1.17-1.45)	Reference	1.24 (1.06-1.42)
		p<0.001	
